# Supplementary material for: Role of dual specificity phosphatase 1 in influencing inflammatory pathways in macrophages modulated by Borrelia burgdorferi lipoproteins
Source: Front Immunol. 2026 Apr 7;17:1766756. doi: 10.3389/fimmu.2026.1766756 (PMC13095537; doi:10.3389/fimmu.2026.1766756)
Supplement: Supplementary file 1 [file DataSheet1.pdf]

Table 1: Genes upregulated in each cluster. Comparative DEG analysis within samples identified the genes that are upregulated in each cluster compared to all other clusters in the study. Genes that are significantly upregulated ($p<0.05$) in each cluster are listed below.

**Cluster 1:** Il31ra, Plau, Fgf13, Fos, Cacna1a, Fat3, Nfkbid, Romo1, Dusp5, Rab7b, Uqcr11, Crip1, Lyzl4, Ndufa2, Dock2, Rasgef1b, Pitpnc1, Dusp4, Rpl41, Nav2, Xylt1, Ndufv3, Atp6v0a1, Hip1, Myo1e, Zdhhc14, Atp5j2, Ifi27l2a, Lncpint, Mef2c, Fosb, H2afj, Hmga2, Uqcr10, Dhrs3, Flna, Arhgap6, Hal, Dapk1, Rpl36, Atp5k
**Cluster 2:** Hilpda, Fgf13, Fat3, Plk2, Rgs2, Phlda1, Pik3cb, Egr2, Tnf, Hmga2, Gramd1b, Prkcb, Pacrg, Junb, Dusp4, Ccnd1, Dnmt3a, Airn, Ckb, Plau, Cdk18, Arhgap25, Mmp12, Pbx1, Mgll
**Cluster 3:** Ms4a4a, Il4ra, Cxcl5, Serpinb2, AW112010, Mxd1, Cxcl3, Mmp13, Mt2, Ifit1, F13a1, Cxcl10, Gbp5, Cmpk2, Isg15, Rsad2, Gbp3, Nos2, Vcan, Mmp14, Ccl5, Gbp2b, Ccl4, Fmnl2, Ifit2, Dst, Ptgs2, Gbp2, Saa3, Ifit3, Ccr1, Il15, Ptges, Il1rn, Slfn8, Socs3, Thbs1, Sdc4, Il1a, Sod2, Cebpb, Nupr1, Rnf213, Slc28a2, Ifi204, Nlrc5, Ifi203, Ms4a6d, Herc6, Oasl1, Slc9a3r1, Il1b, Notch1, Slfn5, Stat2, Acod1, Pfkfb3, Gbp7, Rai14, Dtx3l, Hdac9, Parp14, Mndal, Ddx60, Ezr, Usp18, Cd14, Ch25h, Cemip2, Daxx, Sp140, Clmp, Aoah, Cept1, Fcgr2b, Ifi211, Timp1, Zup1, Pnp, Sbno2, Nampt, Hif1a, Plagl2, Marcks, Ifi202b, Slc39a14, Cd274, Aff1, Mt1, Jak2, Stat3, Sdc1, Slfn9, Etnk1, Ifi44, Mdm2, Tor1aip2, Filip1l, Pdgfa, Rtp4, B2m, Tmtc2, Ms4a6c, N4bp1, Samhd1, Stat1, Parp9, Zfp36, Sipa1l1, Oasl2, Ccl3, Fam241a, Stk40, Trim25, Sat1, Pla2g4a, Atxn1, Ifih1, Sppl2a, Clec2d, 45352, Sp100, Trim30a, Hk2, Il17ra, Lgmn, Eif2ak2, Lrch1, Fcgr1, Adap2, Tiparp, Usp25, Clec4e, Fndc3a, Tlk2, Ptpn1, Wfdc17, Cxcl16, Mcoln2, Samd9l, Slc7a2, Btg1, Susd6, Cd40, Serp1, Mmp19, Nr3c1, Mgst1, Peli1, Errfi1, Ddx58, Tlr2, Ikzf1, Tor1aip1, Ggta1, Bach1, Xrn1, Glrx, Atxn7l1, Casp4, Selenop, Hck, Tab2, 45356, Il10, Camkk2, Aftph, Epsti1, Ifi207, Ascc3, Ifitm1, Mir155hg, Map2k1, Akt3, Lcp2, Ldlr, Znfx1, Arf5, Cacna1d, Etv3, Exoc6b, Stx11, Ankrd17, B3gnt2, Xdh, Gna13, Prr13, H3f3b, Dock4, Rab31, Cd47, Ranbp2, Slc7a11, Tor3a, Marcksl1, Tapbp, Prdx5, Smg1, Dram1, Zc3h7a, Snx10, Irf7, Tax1bp1, Lgals9, Tmbim6, Spen, Sgk3, Map4k4, Dennd1a, Ccrl2, Dusp16, Vmp1
**Cluster 4:** Ch25h, Pla2g4a, Oasl1, Nos2, Ccl5, 45352, Serpine1, Timp1, Saa3, Usp18, Gbp5, Cxcl3, Herc6, Fmnl2, Nlrc5, Nupr1, Vcan, Rsad2, Il15, Cmpk2, Slc28a2, Ddx60, Stat2, Ccl4, Ifit2, Ifit1, Il4ra, Zup1, Rai14, Cept1, Socs3, Rnf213, Cxcl10, Gbp2b, Mmp14, Gbp2, Etnk1, Hdac9, Sipa1l1, Cacna1d, Cxcl5, Pfkfb3, Fam241a, Mt2, Ikzf1, Acod1, Cd40, Cebpb, Parp14, Cd274, Peli1, Gbp3, Dtx3l, Aoah, Gbp7, Ifih1, Pdgfa, Sbno2, Isg15, Dram1, Daxx, Slfn8, Stat1, Hck, Epsti1, Eif2ak2, Oasl2, Ccl3, Adap2, Xrn1, Slfn9, Ddx58, Sp100, Slc39a14, Ifi204, Slfn5, Cemip2, Hif1a, Ifi203, Plagl2, Ifi44, Slc7a11, Ms4a6d, Aff1, Jak2, Tor1aip2, Dst, Parp9, Lrch1, Filip1l, Sp140, Sod2, Notch1, Samhd1, Ezr, Fndc3a, Slc7a2, Tlr2, Ifi211, Clmp, Ifi202b, Xdh, Trim25, Sgk3, Stat3, Ptges, Trim30a, Bcl2a1d, Marcks, Clec2d, Tnfrsf11a, Atxn1, Exoc6b, Ptgs2, Usp25, Camkk2, N4bp1, Mob3b, Rab31, Fnbp1l, Ifit3, Ankrd17, Map2k1, Rtp4, Sdc1, Zfp36, Mndal, Nampt, Tcf4, Aim2, Fcgr2b, Ccr1, Susd6, B3gnt2, Mmp13, Znfx1, Samd9l, Etv3, Chst11, Tiparp, Spp1, 45356, Sdc4, Mcoln2, Clec4e, Arf5, Mxd1, Ccl2, Sppl2a, Akt3, Larp1, B2m, Aftph, Slc9a3r1, Tor3a, Morrbid, Eml4, Tlk2, Cd14, Il17ra, Nr3c1, Actr2, Tab2, Synj1, Ext1, Nek6, mt-Nd1, Hipk2, Dennd1a, Plpp3, Cxcl16, Ggta1, Fcgr1, Msr1, Ascc3, Srgap2, Anpep, Pfn1, Notch2, Selenof, Snx10, Ptpn1, Diaph2, Bcl2a1b, Stk40, Akap13, Lgmn, Sem1, Nsd3, Tmtc2, AW112010, Parp8
**Cluster 5:** Id3, Egr3, Ccl7, Slc9a9, Nr4a3, Cd86, Zfp36l1, Nr4a2, Arhgap15, Egr1, Apoe, Ccrl2, Fosb, C1qa, Arl15, Atrnl1, Ier3, Cd74, Eepd1, Man1a, Arhgap24, Trib1, Ifitm1, Osbpl8, C1qc, Dusp1, Bcas3, Rcbtb2, Clec4n, Rgs1, Fcgrt, Mertk, Dennd4a, Fnip1
**Cluster 6:** Ifitm1, Lst1, Fosb, Ngp, Aif1, Cd74, Slc9a9, Fcgrt, Apoe, Nr4a2, S100a8, S100a9, Atrnl1, Egr3, Thbs1
**Cluster 7:** Hist1h1b, Cenpf, Hist1h2ap, Prc1, Top2a, Nusap1, Hist1h2ae, Hist1h4d, Cenpe, Pclaf, Birc5, Cdca8, Diaph3, Hist1h1e, Tpx2, Stmn1, Smc2, Incenp, Atad2, Hmgb2, Tmpo, Rad51b, S100a9, Rrm1, S100a8, Ezh2, Smc4, Mki67, Ngp, Tuba1b, Tubb4b, Mcm6, Dnmt1, Tubb5, Nasp, Ckap5, Hist1h1c, Rad21, Ccnd1, Ube2s, Hmgb1, Nsd2, Fam111a, Dek, Anp32b, Nucks1, Smc1a, Ranbp1, Ran, Nap1l1, H2afz, Anp32e, Csf1, Snrpd1, Baz1b, Hnrnpab, Arl6ip1
**Cluster 8:** Egr3, Mertk, Cd86, Nr4a3, Slc9a9, Nr4a2, Man1a, Apoe, Atrnl1, C1qa, Fosb, Ccl7, Eepd1, Zdhhc14, Egr1, Entpd1, Arl15, Arhgap15, Emp1, Rcan1, Fcgrt, Zfp36l1, Trib1, Ier3, Clec4n, Ctsc
**Cluster 9:** Eef1a2, Mgll, Mfge8, Atp6v0d2, Dnmt3a, Itgax, Ckb, Ppdpf, Ctsk, Mmp19, Arhgap25, Tspan5, Fn1, Pacrg, Prkcb, 1110008P14Rik, Hvcn1, Myo1f, Acp5
**Cluster 10:** Ctsk, 45352, Mmp12, Slc28a2, Eef1a2, Ifit2, Cmpk2, Ddx60, Rsad2, Fn1, Ifit1, Itgax, Txnip, Ifih1, Samhd1, Gbp5, Sdc1, Mgll, Herc6, Cd274, Atp6v0d2, Bcl2a1b, Cept1, Mfge8, Gbp2b, Rnf128
**Cluster 11:** Hist1h1b, Nusap1, Hist1h2ap, Hist1h2ae, Pclaf, Top2a, Hist1h1e, Birc5, Prc1, Cenpe, Smc2, Cenpf, Diaph3, Cdca8, Tpx2, Serpine1, Smc4, Rrm1, Hist1h4d, Tmpo, Stmn1, Ccl2, Incenp, Mki67, Cxcl10, Timp1, Nos2, Hmgb2, Tuba1b, Ckap5, Hist1h1c, Tubb5, Nucks1, Ube2s

Table 2: Genes upregulated in each sample. Comparative DEG analysis between samples identified the genes that are upregulated in each sample compared to other sample (provided as the title). Genes that are significantly upregulated ($p<0.05$) in each cluster are listed below.

**Uninfected vs LP4**:<br>Egr1, Fos, Plau, Rasgef1b, Dusp5, Jun, Snx29, Zdhhc14, Pitpnc1, Dennd4a, Cacna1a, Lncpint, Rgs1, Il31ra, Rab7b, Slc9a9, Fat3, Xylt1, Slc16a10, Dusp4, Mtss1, Nav2, Arhgap15, Mef2c, Jmjd1c, Maml3, Fgf13, Fnip1, Sgms1, Dock2, Pde7b, Snx24, Vav3, Ier2, Mafb, Snx8, Dapk1, Cadm1, Cmss1, Plk2, Dock10, Cxcl2, Dhrs3, Lpin2, Tnf, Nlrp3, Peak1, Asap1, Plaur, Cd83, Gramd1b, Runx1, Airn, Antxr2, Fnbp1, Rbm47, St3gal5, Itpkb, Myo1e, Arhgap10, Atp6v0a1, Nfam1, St3gal1, Zbtb20, Ptpn18, Bcl2l11, Arhgap6, Plekho2, Pbx1, Ndufv3, Dgkz, Hmga2, Gm42418, Ifrd1, Osbpl8, Man1a, Lrmda, Zswim6, Nfe2l2, Neat1, Camk1d, Lars2, Dnmt3a, Fnip2, Dusp1, Cdk8, H2afj, Fam20c, Tmem164, Trps1, Ubc, Slc43a2, Hal, Piezo1, Rpl36, Map3k5, Wwox, Rps15, Rabgef1, Ndufb7, Uqcr11, Lyzl4, Gripap1, Myo1f, Ndufs7, Cd44, Romo1, Atp13a2, Rpl41, Erp29, Sema4d, Dleu2, Gapdh, Spag9, Ndufa11, Slc38a6, Atp5j2, Myo9b, Rpl37, Fbxo11, Slc37a2, Ndufa2, Uqcr10, Wwp1, Itgav, Ndufs6, Rps29, Sestd1, Timm10b, Epb41l2, D8Ertd738e, Crip1, Rpl31, Atp8b4, Ap2s1, Ptpra, Stap1, Tpm1, Lyst, Tyrobp, Rps21, Atp5k, Cbl, Ncf1, Plekho1, AY036118, Cd84, Gphn, Gas7, Ankrd33b, Tgfb1, Csf1r, Fau, Cmip, Colec12, Apoe, Rpl37a, Rps26, Atox1, Tmem160, Irak2, Cops9, Uqcrq, Tmem256, Sertad2, Exoc4, Tln1, Top1, Pgls, Soat1, Scand1, Igf1, Hivep2, Rpl28, 0610012G03Rik, Mif, Specc1, Ndufa13, Rpl18a, Ucp2, Syk, Gadd45b, Lgals1, Syngr1, Zc3h12c, Metap2, Ano6, Ifi27l2a, Rel, Apbb2, Rpl35, Fermt3, Cox4i1, Tnfaip8, Lamtor4, Ubl5, Nsa2, Rpl10a, Pstpip2, Rtf2, Wasf2, Large1, Rrp1, Rpl26, Ptms, Sms, Eno1, Itga5, Ubap2l, Nav1, Mef2a, Map4k3, Cdc37, Trem2, Plekhm3, Capns1, Rpl38, Nfkbiz, S100a1, Pam, Elob, Rplp1, Abca1, Emp1, Actb, Fxyd5, Mapre2, Ctsd, Coro1a, Abr, Cd52, Vps13c, Ier3, Flna, Cox5b, Tcf25, Slc41a2, Ndufs5, Mtch1, Gm10076, Emp3, Rps14, Gm26917, Gpi1, Csf2ra, Sf3b2, Gstm1, Ptpre, Fuca1, Pfdn5, Fabp5, Rps12, Pld4, Atp5e, Rps5, Nckap1l, 1810058I24Rik, Tle5, Zeb2, Capg, Timm13, Aprt, Coro1c, Ubb, Elk3, Nfat5, Rps11, Lsp1, Hint1, Rpl36a, Hmgb1, Rps8, Atpif1, Msrb1, Nceh1, Rrbp1, St13, Cox7a2l, Ctsa, Fam129b, Lrrfip2, Aldh2, Atp5g1, Hsp90ab1, Lilr4b, Kcnn4, Ccnl1, Rpl10, Rps20, Rps17, Arhgap31, Atp5o, Cyba, Atp5d, Mitf, Gpnmb, Dot1l, Prelid1, Dhrs9, Lmna, Rps19, Rplp2, Cd68, Chmp2a, Mbnl2, Atp6v0d2, Pltp
**LP1 vs Uninfected**:<br>Cebpb, Gnb2, Pfn1, Ccl4, Btf3, Card19, Lgmn, Atp5g2, Sod2, Rpl9, H2-D1, Cd14, Junb, Ppia, Ccl3, Atp6v0e, Hilpda, Unc93b1, mt-Atp6, Ran, Cxcl1, mt-Cytb, Pcna, mt-Co2, Spp1, Snx3, H3f3a, Slc25a5, mt-Nd4
**LP4 vs Uninfected**:<br>Nos2, Cxcl3, Ifit1, Cmpk2, Cxcl10, Rsad2, Gbp5, Ccl5, Slc28a2, Ccl4, Il4ra, Ifit2, Cebpb, Fmnl2, Vcan, Saa3, Herc6, Il15, Gbp2b, Socs3, Timp1, Oasl1, Ddx60, Nlrc5, Isg15, Usp18, Gbp2, Pla2g4a, Mmp13, Stat2, Mt2, Rnf213, Sod2, AW112010, Zup1, Ccr1, Pfkfb3, Rai14, Etnk1, Cd274, Ms4a6d, Ifi204, Acod1, Ccl3, Parp14, Ifi203, Slfn8, Fam241a, Dst, Daxx, Arf5, Pdgfa, Sdc1, Slfn5, Cd14, Ifih1, Ifit3, Peli1, Ikzf1, Ptgs2, Cemip2, B2m, Lgmn, Tor1aip2, Ezr, Ms4a4a, Ptges, Samhd1, Aff1, Sbno2, Cd40, Zfp36, Plagl2, Sdc4, Bcl2a1d, Slc7a2, Jak2, Ifi202b, Hif1a, Lrch1, Mndal, Epsti1, Filip1l, Eif2ak2, Nampt, Ifi44, Stat1, Oasl2, Aoah, Ifi211, Marcks, Cacna1d, Slc7a11, Adap2, Sp140, Stat3, Sp100, Fndc3a, Il1rn, Clmp, Selenof, Gnb2, Xrn1, Trim30a, Ccl2, Ddx58, Dram1, Pfn1, Srp9, Hck, Pnp, 45356, Usp25, Card19, Trim25, Clec2d, Bcl2a1b, Map2k1, N4bp1, Atxn1, Sppl2a, Rab31, Bst2, Tlr2, Samd9l, Sat1, Cstb, Aftph, Abracl, Xdh, Mob3b, Ankrd17, Susd6, Akt3, Tlk2, H2-D1, Mdm2, Nr3c1, Sem1, B3gnt2, Clec4e, Btf3, Tiparp, Mcoln2, Il1a, Chst11, Tmbim6, Tor3a, Il17ra, Txn1, Ptpn1, Exoc6b, Ascc3, Plpp3, Znfx1, Etv3, Larp1, Tab2, Casp4, Tcf4, H3f3b, Il1b, Snx10, Ms4a6c, Hipk2, Cxcl16, Aim2, Btg1, Pcbp2, Unc93b1, Prr13, Glrx, mt-Co2, Spred1, Dennd1a, mt-Cytb, Capza2, Fcgr1, Eml4, Mbd2, Ext1, Nek6, Parp8, mt-Atp6, Pcna, Actr2, Ctsl, Bach1, Ldlr, Tor1aip1, Slc6a6, Dazap2, mt-Nd1, Spp1, Hnrnpk, mt-Nd4, Ifi207, Morrbid, Txnip, Gna13, Pabpc1, Zc3h7a, Srgap2, Sumo2, Diaph2, Synj1, Vmp1, Nsd3, Snx3, Srxn1, Rpl9, Ranbp2, Cd47, Tnfrsf11a, H3f3a, Tapbp, Fnbp1l, Mt1, Smg1, Arf1, Vapa, Mir155hg, Atxn7l1, Hmgcs1, Marcksl1, Hnrnpa2b1, Esyt1, Cd53, Lgals9, Tax1bp1, Rala, Denr, Resf1, Anpep, Ppp2r5a, Atp5g2, Notch2, Msr1, Mmp12, Akap13, Irf2, Sdcbp, St8sia4, Zc3hav1, Rbm3, Ptafr, Galnt7, Ptbp3, Ppp4r2, Atp6v0e, Prdx1, Ube2d3, Slc25a5, Pla2g7, Prdx5, Gsap, Rin2, Gls, Tbc1d9, Ran, Spen, Rhob, S100a10, Gch1, Tnfrsf1b, Creb5, Lpp, Fabp4, Ppia, Sh3bgrl, Rab11fip1, Dusp16, Eif4g2, Ube2e2, Pid1, Atp5g3, Cers6, Basp1, B4galt5, Med13l, Alpk1, Tmsb4x, Cd9, Brd2, Foxp1, F10, Sh3glb1, Txndc17, Apobec1, Rpl22l1, mt-Nd2, Wdr1, Adam8, Rpl7a, Ap3b1, Ctsc, Pik3ap1, Esd, Ostf1, Rassf4, Cul3, Eif2s2, H2-K1, Cap1, Tut7, Csf2rb, Cdc42, Laptm4a, Kpna4, Cggbp1, Tnip3, Tomm20, Hnrnpf, Tnip1, Bmp2k, Wfdc17, Snd1, Ptges3, Adgre1, Mtpn, Dab2, Pcbp1, Txnrd1, Kpna3, Sptlc2, Vti1a, Gng12, Lamp2, Ube2d2a, Eif4g3, Ifnar1, Serinc1, Dnaja2, Lyn, Csnk1a1, Ilrun, Ptprj, Psma3, Itgam

Table 3: Primers used in this study. Following are the primers used for the qPCR analysis.

Actb-F	GAC TCA TCG TAC TCC TGC TTG
Actb-R	GAT TAC TGC TCT GGC TCC TAG
Cxcl1-F	GTG CCA TCA GAG CAG TCT
Cxcl1-R	CCA AAC CGA AGT CAT AGC CA
Cxcl2-F	CTT TCC AGG TCA GTT AGC CTT
Cxcl2-R	CAG AAG TCA TAG CCA CTC TCA AG
Dusp1-F	GAA CAA ACA CTC TCC CTC CAG
Dusp1-R	CAC TAC CAG TAC AAG AGC ATC C
Clec4d-F	CAG TTT CAC CAC ACT TCC TCT
Clec4d-R	CTG ATC CCT TGC GTC TTC G
Gbp2-F	GAT GGC ACC AAC ATA GGT CTG
Gbp2-R	GCA GAA TTC ACC TCA TAC ATC TTG
Gbp5-F	TGC TAC TGA GGA GGA TTG CTA
Gbp5-R	GGA TCT GGA TGT GGT GTG TG
Nlrp3-F	CGG TTG GTG CTT AGA CTT GA
Nlrp3-R	CAC TCA TGT TGC CTG TTC TTC
ccl5-F	CCT CTA TCC TAG CTC ATC TCC A
ccl5-R	GCT CCA ATC TTG CAG TCG T
Ifit1-F	TGA AGC AGA TTC TCC ATG ACC
Ifit1-R	GCA AGA GAG CAG AGA GTC AAG
Isg15-F	CCC CCA TCA TCT TTT ATA ACC AAC
Isg15-R	CAC AGT GAT CAA GCA TTT GCG
Il1b-F	CTC TTG TTG ATG TGC TGC TG
Il1b-R	GAC CTG TTC TTT GAA GTT GAC G
Cebpb-F	CCG CAG GAA CAT CTT TAA GTG A
Cebpb-R	GTT TCG GGA CTT GAT GCA ATC
Il1f9-F	CAG GTG TGG ATC TTT CGT AAT CA
Il1f9-R	CAT GGG AGG ATA GTC ACG CTG
Rsad2-F	ACG CCA ACA TCC AGA ATA GAC
Rsad2-R	CCA GAA GAT GAA AGA CTC CTA CC
Ccl4-F	CAA CTC CAA GTC ACT CAT GTA CT
Ccl4-R	GGA TTA CTA TGA GAC CAG CAG TC
Hcar2-F	GAG TAG ATG TCA CAG TTG CGT
Hcar2-R	TCA TTT GCT TCC TAC CCA GTG
Ccrl2-F	TGT TGT CCA GGT AGT CGT CTA
Ccrl2-R	ATC AAG CAA CCT GCC TCA A

Fig. 1: Extraction and antigenicity characterization of lipoproteins from Borrelia. A) Lipoproteins were purified from B31 strain of *Bb* using Triton X-114and the proteins in different fractions were visualized by coomassie staining of SDS-PAGE gel [Lanes: 1-Ladder, 2-insoluble fraction (Pellet), 3-water soluble fraction (aqueous) and 4-Triton X-114 soluble fraction (Lipoproteins)]. Immunoblots showing the reactivity of three fractions of Bb proteins against B) *Bb*FlaB antibodies showing the purity of the purified lipoproteins and C) B31-strain of *Bb* infected mouse serum showing the antigenicity of the purified lipoproteins. Molecular weight of the ladder proteins mentioned on the left side of each figure and the fraction details are mentioned at the top of each lane.

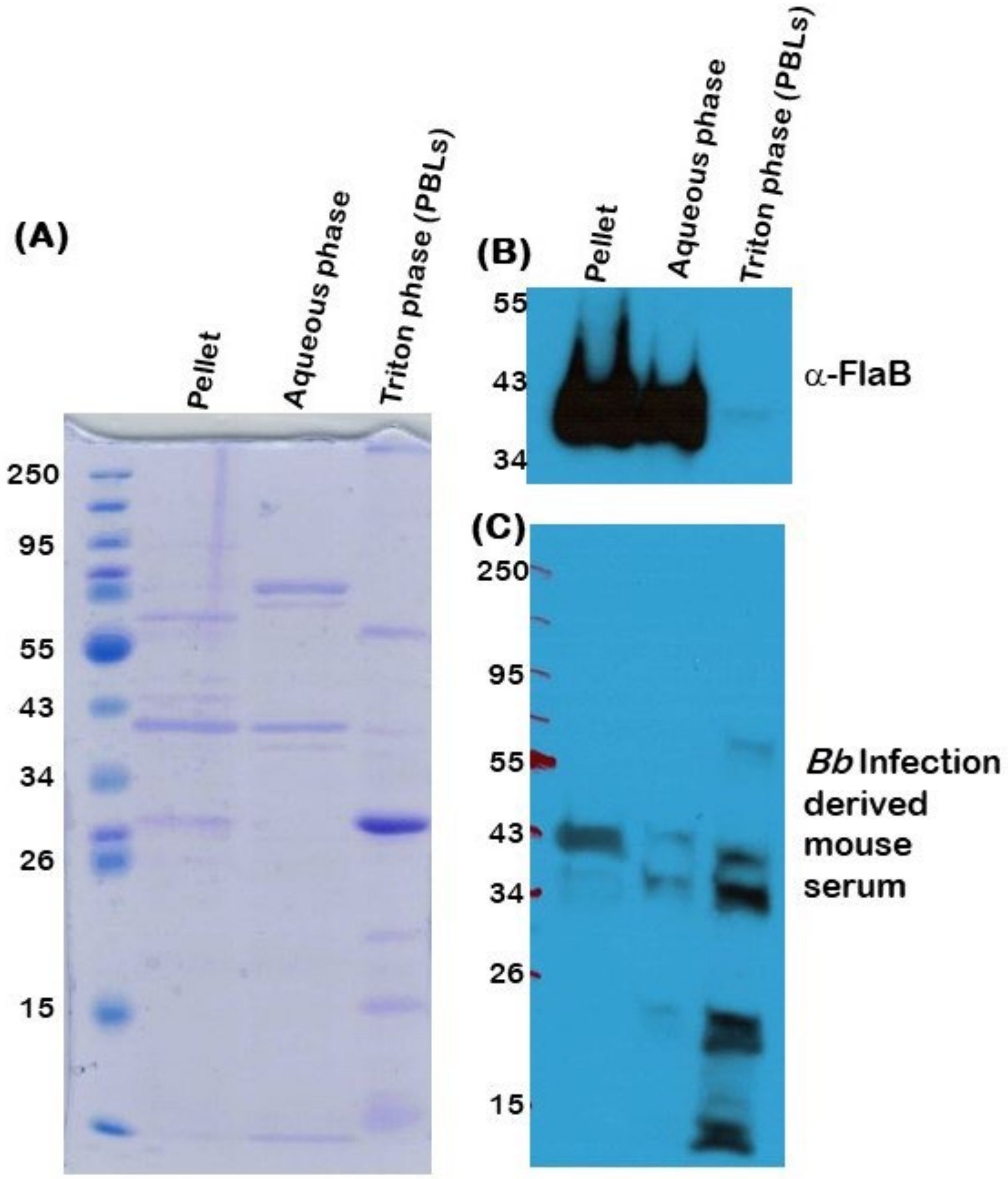

Fig 2: scRNAseq analysis of Borrelial lipoprotein infected BMDM. Total BMDM barcodes were subjected to cell count analysis and the a) tSNE plot and b) UMAP plots showing three separate clusters of control BMDM and lipoprotein treated BMDM at 1 hpi and 4 hpi. c) tSNE plot showing the clusters of Ptprc+Actb+ barcodes within each sample. d) Violin plots showing the log2 expression and cellular distribution of the leukocyte marker gene, Ptprc and macrophage marker gene, Adgre1. e) Heatmap showing the DEGs among the three clusters of BMDM and the putative names of each cluster along with the putative functions were mentioned over the respective blocks. f) Venn diagram showing the distribution of clusters between the three samples. g) Heatmap showing the DEGs between all the identified clusters and the putative functions are represented over each set of genes. h) Violin plots showing the log2 expression and cellular distribution of M1 and M2 macrophages specific genes among the eleven clusters. i) tSNE plots showing the expression of selected genes involved in inflammation, antigen presentation, cell cycle and M2 polarization, colour scale represent the log2 expression level of genes.

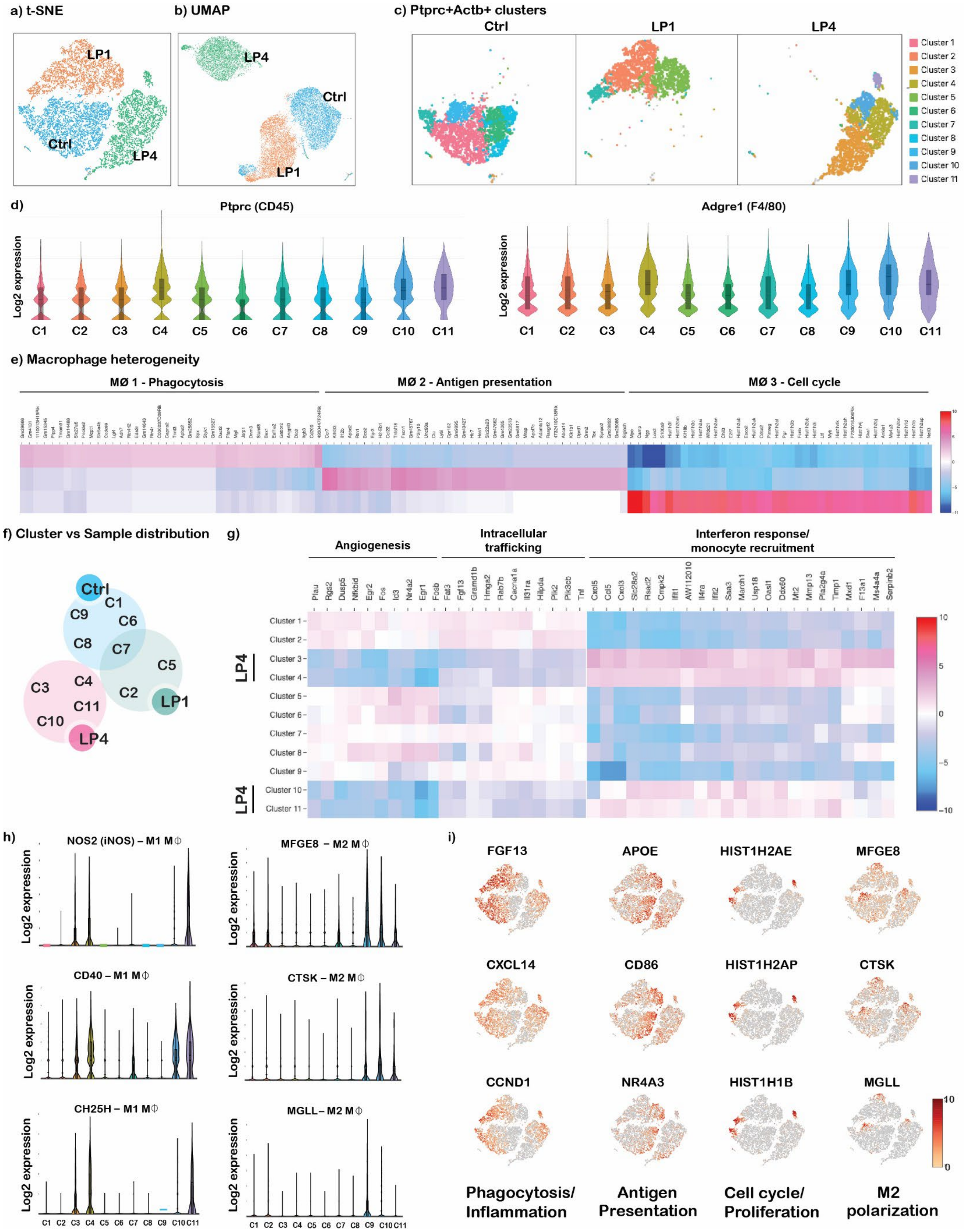

a) t-SNE
LP1
Ctrl
LP4
b) UMAP
LP4
Ctrl
LP1
c) Ptprc+Actb+ clusters
Ctrl
LP1
LP4
Cluster 1
Cluster 2
Cluster 3
Cluster 4
Cluster 5
Cluster 6
Cluster 7
Cluster 8
Cluster 9
Cluster 10
Cluster 11
d)
Ptprc (CD45)
Adgre1 (F4/80)
Log2 expression
C1 C2 C3 C4 C5 C6 C7 C8 C9 C10 C11
e) Macrophage heterogeneity
MØ 1 - Phagocytosis
MØ 2 - Antigen presentation
MØ 3 - Cell cycle
f) Cluster vs Sample distribution
Ctrl
C1 C9 C6 C8 C7 C5 C3 C4 C2 C11 C10
LP1
LP4
g)
Angiogenesis
Intracellular trafficking
Interferon response/ monocyte recruitment
Plau Rgs2 Dusp5 Nfkbid Egr2 Fos Id3 Nr4a2 Egr1 Fosb Fat3 Fgf13 Gramd1b Hmga2 Rab7b Cacna1a Il31ra Hilpda Plk2 Plk3cb Tnf Cxcl5 Ccl5 Cxcl3 Slc28a2 Rsad2 Cmpk2 Ifit1 AW112010 Il4ra Ifit2 Saa3 March1 Usp18 Oasl1 Ddx60 Mt2 Mmp13 Pla2g4a Timp1 Mxd1 F13a1 Ms4a4a Serpinb2
Cluster 1
Cluster 2
Cluster 3
Cluster 4
Cluster 5
Cluster 6
Cluster 7
Cluster 8
Cluster 9
Cluster 10
Cluster 11
LP4
LP4
10
5
0
−5
−10
h)
NOS2 (iNOS) – M1 MΦ
MFGE8 – M2 MΦ
CD40 – M1 MΦ
CTSK – M2 MΦ
CH25H – M1 MΦ
MGLL– M2 MΦ
Log2 expression
C1 C2 C3 C4 C5 C6 C7 C8 C9 C10 C11
i)
FGF13
APOE
HIST1H2AE
MFGE8
CXCL14
CD86
HIST1H2AP
CTSK
CCND1
NR4A3
HIST1H1B
MGLL
10
0
Phagocytosis/ Inflammation
Antigen Presentation
Cell cycle/ Proliferation
M2 polarization

Fig 3: Gene expression pattern of Borrelial lipoprotein infected BMDM. tSNE plots showing the log2 expression and cellular distribution of the identified genes. Sample information of each tSNE plot was represented at the bottom of the plots and the corresponding genes are represented at the left of each plot. Scale at the right end represent the expression levels of each gene, where red represent the highest expression and grey represent the lowest expression.

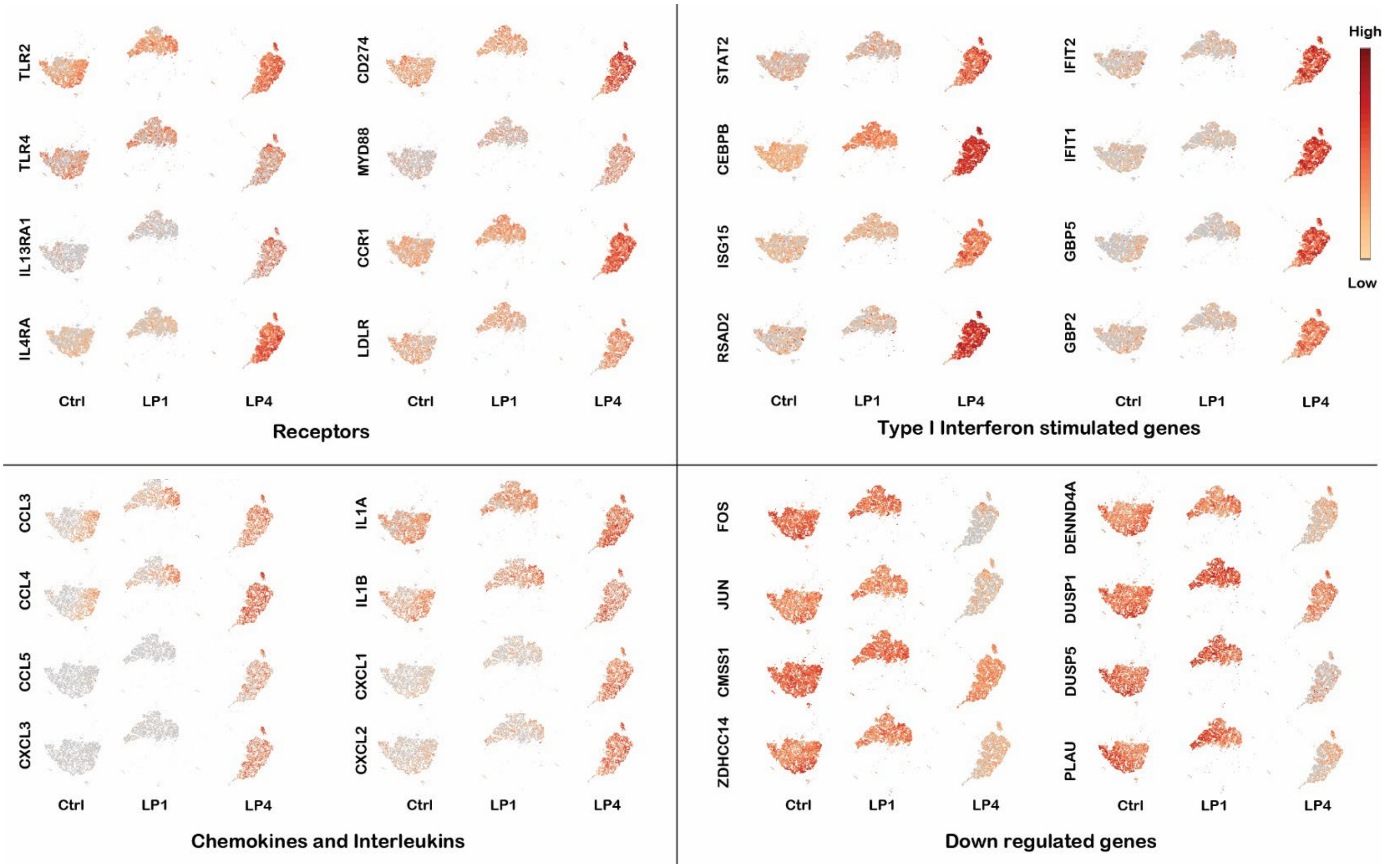

Fig 4: Pseudotime analysis revealing *Bb*LP induced transcriptional changes in BMDM: Harmony integrated UMAPs were generated using Seurat and Pseudotime analysis was performed using monocle3. a) UMAP plot showing the pseudotime analysis split by clusters, where the clusters are identified as numbers 1-13. The trajectories are represented as lines and the stages of the BMDM are represented as numbers within the circle, where smaller numbers (0-5) indicate early stages, while larger numbers (6-20) denotes advanced stages in macrophages. b) UMAP plot showing the clusters split by samples, where the samples are marked as Ctrl, LP1 and LP4. These plots revealed that macrophages in Ctrl samples showed smaller numbers (0-5) denoting early stages, while macrophages in LP1 showed medium numbers (6-12) and LP4 showed larger numbers (6-20).

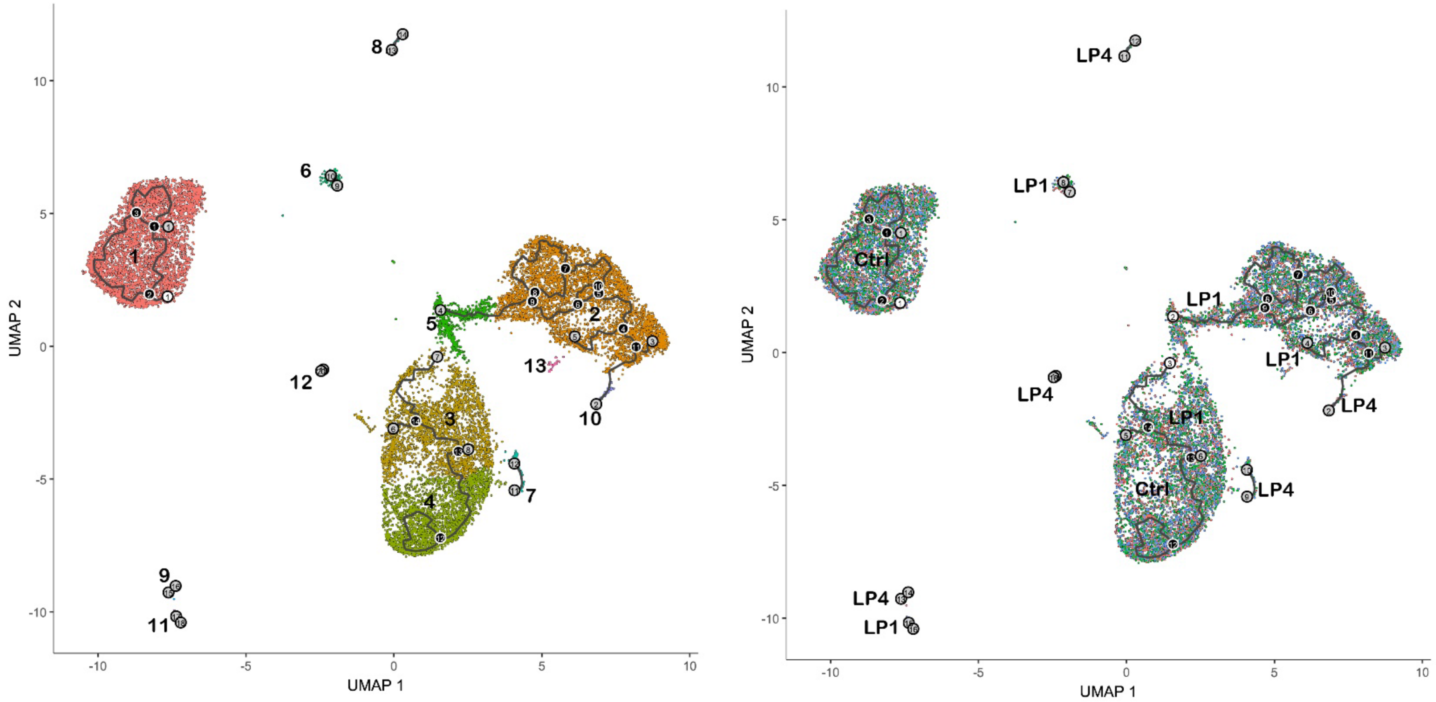

Fig 5a: KEGG pathway of upregulated genes from Ctrl. KEGG pathway analysis of the identified upregulated genes in Control. Each red bar represents the number of genes involved in each pathway as -Log10 P value and the corresponding pathway information of each bar is mentioned at the left of each bar.

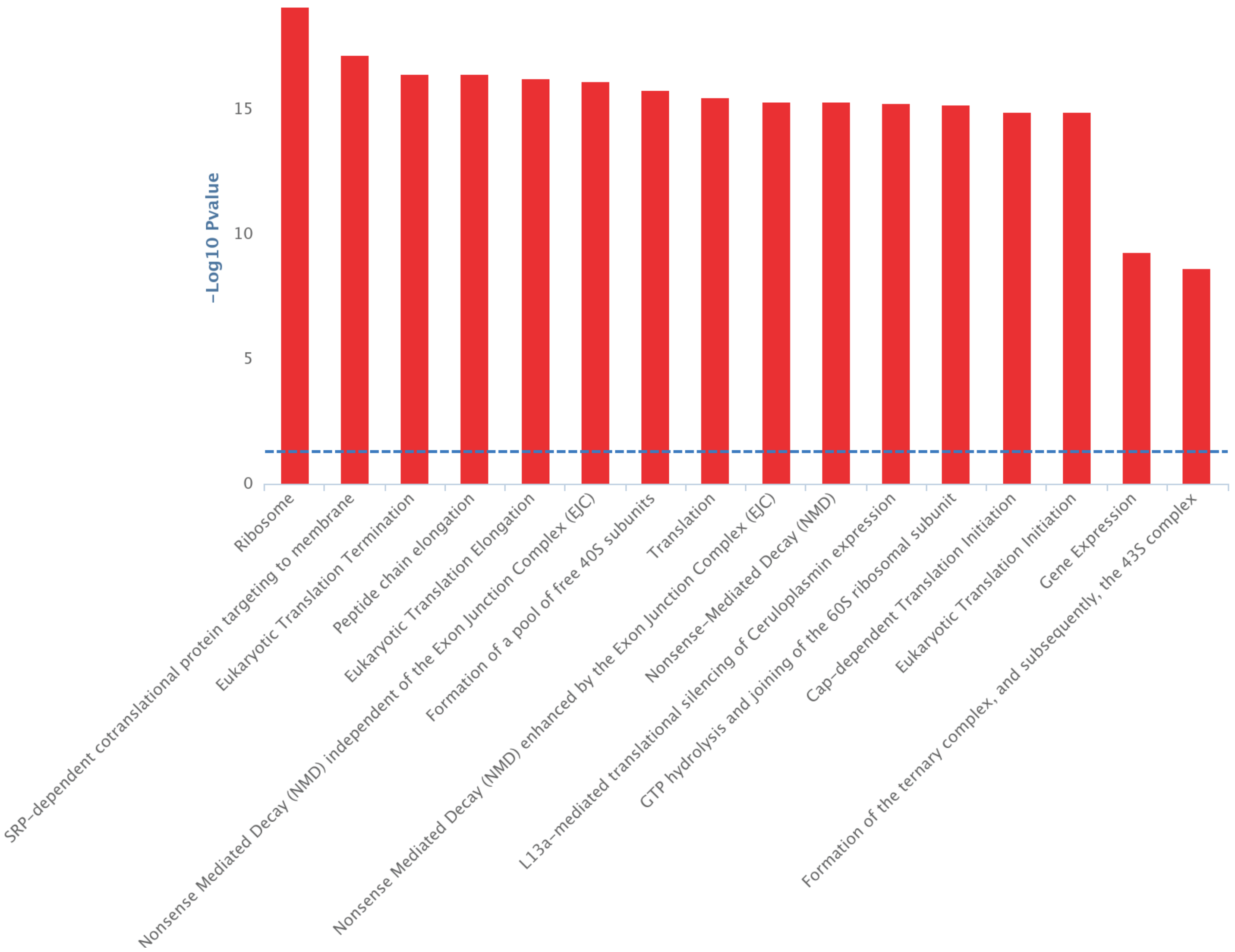

Fig 5b: KEGG pathway of upregulated genes from LP1. KEGG pathway analysis of the identified upregulated genes in LP1. Each red bar represents the number of genes involved in each pathway as -Log10 P value and the corresponding pathway information of each bar is mentioned at the left of each bar.

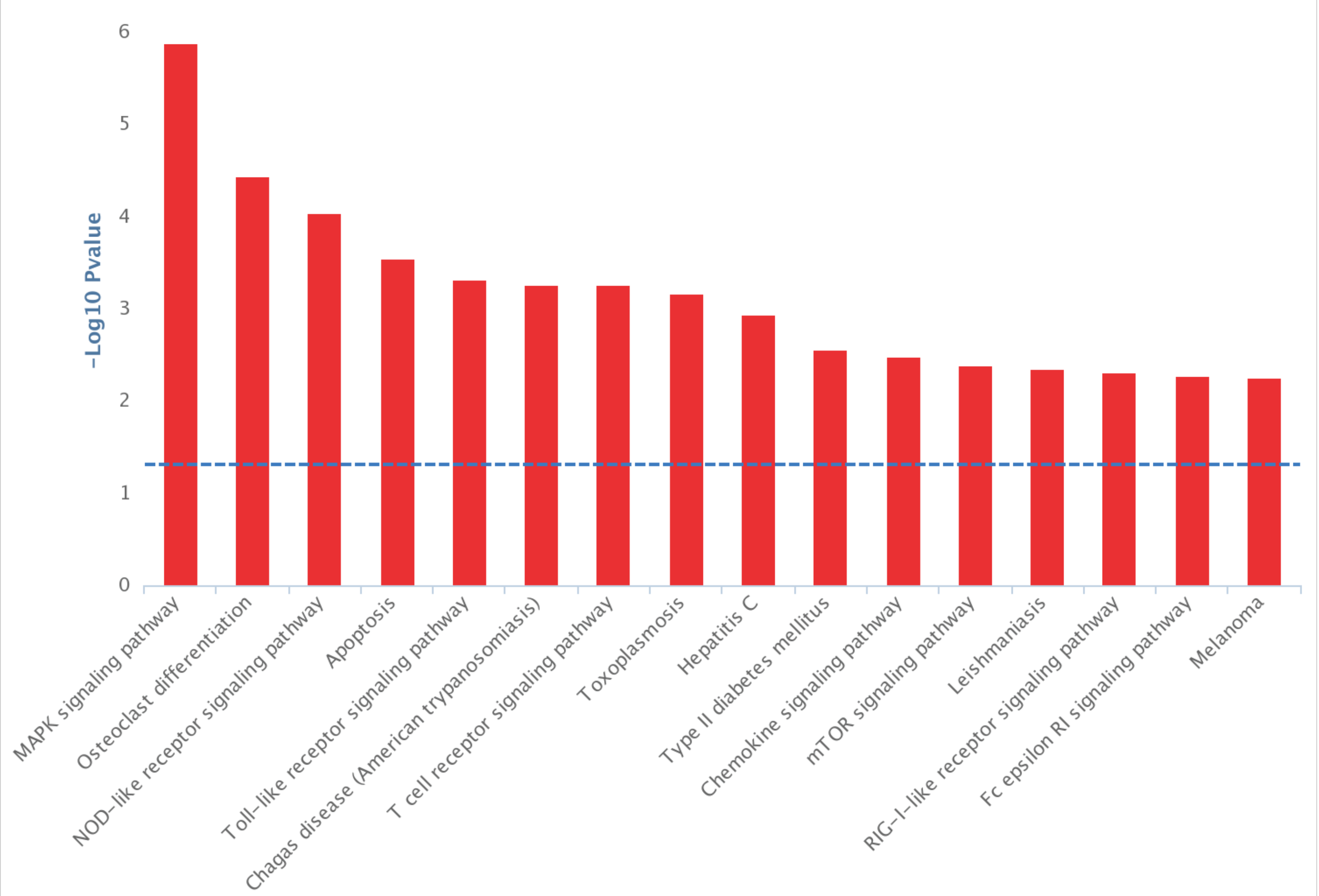

Fig 5c: KEGG pathway of upregulated genes from LP4. KEGG pathway analysis of the identified upregulated genes in LP4. Each red bar represents the number of genes involved in each pathway as -Log10 P value and the corresponding pathway information of each bar is mentioned at the left of each bar.

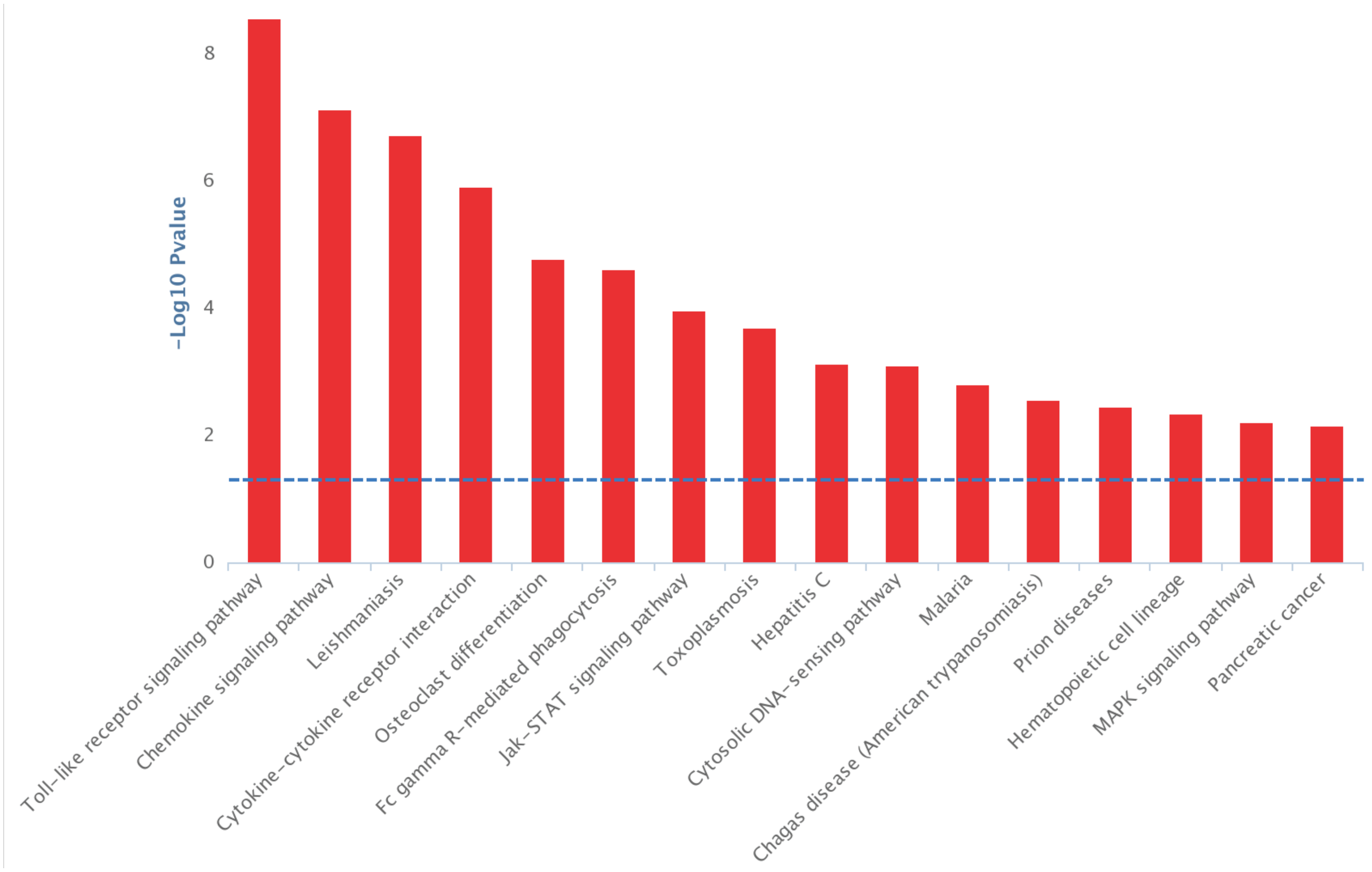

-Log10 Pvalue
0
2
4
6
8
Toll-like receptor signaling pathway
Chemokine signaling pathway
Leishmaniasis
Cytokine-cytokine receptor interaction
Osteoclast differentiation
Fc gamma R-mediated phagocytosis
Jak-STAT signaling pathway
Toxoplasmosis
Hepatitis C
Cytosolic DNA-sensing pathway
Malaria
Chagas disease (American trypanosomiasis)
Prion diseases
Hematopoietic cell lineage
MAPK signaling pathway
Pancreatic cancer
